# Preparation and Characterization of a Novel Self-Healing Transparent Polyimide Film Based on Dynamic Disulfide Bonds

**DOI:** 10.3390/polym16243461

**Published:** 2024-12-11

**Authors:** Xin Li, Yan Zhai, Kai Yang, Jingjing Bai, Yu Qiu, Yulong Wang

**Affiliations:** Department of Materials Engineering, Taiyuan Institute of Technology, Taiyuan 030008, China; lixin428@tit.edu.cn (X.L.); yangkaicl@tit.edu.cn (K.Y.); baijj@tit.edu.cn (J.B.); qiuyu@tit.edu.cn (Y.Q.); wangyl@tit.edu.cn (Y.W.)

**Keywords:** self-healing, polyimide, disulfide bond, transparent, film

## Abstract

Self-healing optically transparent polyimides have potential applications in optoelectronic device fabrication. In this study, for the first time, we successfully prepared a novel self-healing polyimide film containing reversible disulfide bonds through chemical imidization by introducing cystamine as a self-healing functional monomer into the molecular structure of conventional polyimides. The incorporation of cystamine enabled the films to maintain high transmittance (>87%) and tensile strength (>99 MPa). Meanwhile, tensile tests showed that the prepared film with a cystamine content of 50% achieved an excellent self-healing efficiency of up to 91.8%. Stress relaxation tests further revealed that disulfide bonds were rapidly cleaved upon thermal stimulation and the network topology was rearranged to complete the self-healing process. These results suggest that the dynamic covalent polymer network made of aliphatic disulfide bonds presents a new strategy for the development of optically transparent polyimides with excellent self-healing properties.

## 1. Introduction

With the development of sustainable manufacturing technologies, their application in the fabrication of self-healing materials has attracted considerable interest. Self-healing materials are capable of autonomously detecting damage or defects caused by various changes in the environment and repairing them using external stimulations (such as light, heat, and electricity), thereby increasing their service life, energy efficiency, and decreasing their environmental impacts [1,2]. Due to excellent heat resistance, high light transmission and flexibility, optically transparent polyimides (PIs) have great potential applications in optoelectronic devices such as flexible displays, flexible circuit boards, flexible thin-film solar cells, and artificial skin [3,4]. However, owing to their brittle nature, frequent handling and movements can easily cause mechanical damage (such as scratches and cracks) to flexible devices during use [5,6], leading to the failure of the entire device. Although several strategies have been developed for the self-healing of PIs to recover their normal functions [7,8,9,10], solutions to these problems are still facing more challenges.

Self-healing materials can be classified as extrinsic or intrinsic systems, depending on the mechanism of healing [11]. Extrinsic systems undergo self-healing by releasing polymerizable or cross-linkable agents from capsules or vessels in the matrix [12,13,14]. In contrast, intrinsic systems do not rely on these repairing agents, and they utilize specific and reversible interactions between the components in the matrix [15,16,17]. As an important member of dynamically reversible covalent bonds, disulfide bonds have attracted much attention in the synthesis of self-healing polymers due to the abundance of raw materials, mild reaction conditions, and high responses to a variety of stimuli [18]. Recent studies have shown that introducing functional monomers containing disulfide bonds into conventional polymeric structures can trigger the reorganization of molecular chains through exchange reactions between disulfide bonds, thereby conferring polymers with excellent self-healing properties [19,20,21]. In particular, soft polymeric materials with low glass transition temperatures (T_g_) can be endowed with room-temperature healing properties; however, there are still many challenges facing rigid PIs with high T_g_ [2,22].

Due to strong inter- and intra-molecular charge–transfer (CT) interactions, aromatic PIs have a very rigid molecular structure, which hinders the mobility of polymer chains. Compared with elastomer, it is extremely difficult to exhibit self-healing properties for PIs with higher T_g_ values under mild conditions [23]. Importantly, designing the flexible structures in polymer chains is supposed to improve the chains’ mobility to achieve high self-healing efficiency. For example, long-chain aliphatic diamines, employed as monomers, were incorporated into PIs during synthesis, which could be self-healed at room temperature. However, the acquired material exhibited elastomer-like properties [7]. Recently, semi-aromatic PIs were prepared using aromatic disulfides and long-chain aliphatic diamine monomers with ether linkages; they exhibited a high self-healing efficiency of 98% [24]. However, relatively low T_g_ (<100 °C) and tensile strengths (<60 MPa) limited their use at high temperatures. In addition, the integration of aromatic disulfides often leads to a yellowish appearance and reduced transparency [18,25]. Therefore, the trade-off relationship between self-healing efficiency and other properties (mechanical, thermal, and optical) still needs to be further improved through molecular design.

To overcome these problems, we designed a novel, transparent, self-healing polyimide (SHPI) by copolymerizing cystamine containing aliphatic disulfide bonds with 2,2-bis [4-(3,4-dicarboxyphenoxy) phenyl]propane dianhydride (BPADA) and 2,2-bis [4-(4-aminophenoxy)phenyl]-hexafluoropropanane (HFBAPP). The incorporation of cystamine as a functional monomer provides the active moiety (S-S bonds) capable of restoring the intra-chain bonds in damaged areas and the flexible dianhydride linker in BPADA increases the mobility of polymer chains to enhance the self-healing efficiency [26]. In addition, bulky trifluoromethyl (-CF_3_) in HFBAPP can inhibit the intermolecular charge–transfer interaction, increase the intermolecular free volume, and reduce the stacking of molecular chains, which endows the SHPI films with high transparency. By adjusting the monomer ratio, a transparent SHPI film with both an excellent self-healing capacity and mechanical properties was developed. This study provides a new idea for preparing self-healing polyimides using reversible covalent bonds.

## 2. Materials and Methods

### 2.1. Materials

Cystamine dihydrochloride was purchased from Sigma-Aldrich (Buchs, Switzerland). HFBAPP (purity > 99%) and BPADA (purity > 98%) were purchased from Chinatech Chemical (Tianjin, China). N-methyl-2-pyrrolidinone (NMP, >99.8%) and N,N-dimethylacetamide (DMAc, >99.8%) was purchased from Adamas Reagent (Shanghai, China). Sodium hydroxide, anhydrous sodium sulfate, acetic anhydride, and anhydrous pyridine were purchased from Maclean Biochemical Technology (Shanghai, China). Dichloromethane and methyl alcohol (AR) were purchased from Fuchen Chemical Reagent (Tianjin, China).

### 2.2. Preparation of Cystamine

Cystamine was prepared according to methods described elsewhere [27], with slight modifications. Briefly, cystamine dihydrochloride (6.5 g, 28.87 mmol) and sodium hydroxide (2.31 g, 57.57 mmol) were dissolved in water (10 mL), followed by reaction at room temperature for 1 h with stirring. Cystamine was extracted with dichloromethane (5 × 20 mL). The organic phase was separated and dried over anhydrous sodium sulfate, followed by filtration and drying under vacuum to obtain cystamine as a pale-yellow oily liquid. The chemical structure of cystamine was characterized using FTIR, ^1^H NMR, and ^13^C NMR (Part A in SM).

### 2.3. Synthesis of SHPIs

**Scheme 1:**
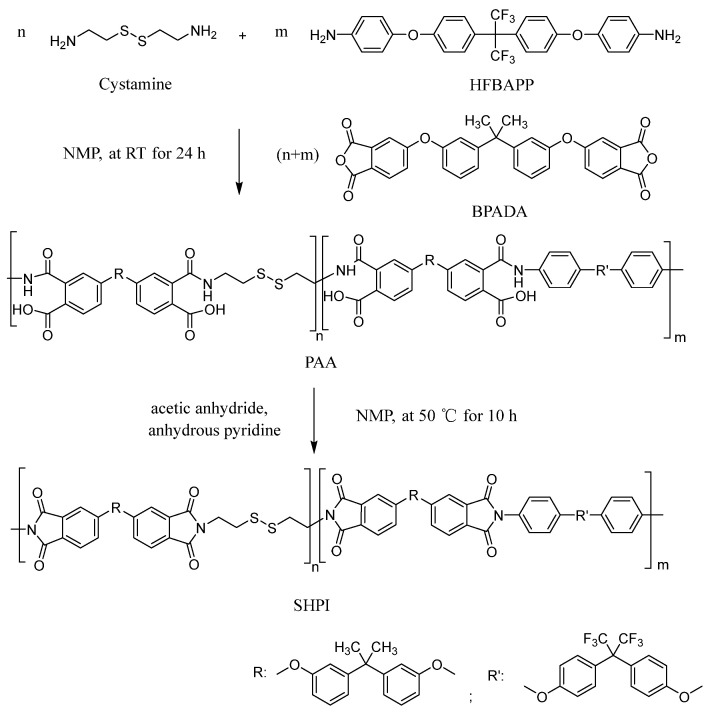
The procedure of synthesizing SHPIs.

SHPIs were synthesized by copolymerizing BPADA with two amines (HFBAPP and cystamine) using a two-step polycondensation method (Scheme 1, Table 1). The molar ratio of BPADA to the total content of HFBAPP and cystamine was maintained at 1:1. The molar ratios of HFBAPP to cystamine were set as follows: 10:0, 9:1, 8:2, 7:3, 6:4, 5:5, and 4:6. First, BPADA (2.602 g, 5 mmol) and NMP (17 mL) were added to a flask and stirred continuously under an N_2_ atmosphere until the BPADA was completely dissolved. Then, a certain amount of HFBAPP was added and stirred until complete dissolution. Third, a mixture of cystamine and NMP at corresponding molar ratios were added. In the reaction, the total solids content was 18 wt%. The mixed solution was continuously stirred at room temperature for 24 h to obtain a viscous polyamic acid (PAA) solution. After adding acetic anhydride (1.17 mL, 12.5 mmol) and anhydrous pyridine (1.00 mL, 12.5 mmol), the reaction system was maintained at 50 °C for 10 h to synthesize SHPIs via chemical imidization. After slowly transferring the solution above into 200 mL of methanol/water (1:1, *v/v*), SHPIs were precipitated and collected by filtration, followed by washing three times with methanol and drying overnight at 70 °C under vacuum.

### 2.4. Preparation of SHPI Films

The SHPI films were prepared by casting a 20% (*w*/*v*) solution of SHPIs in DMAc onto a fused silica substrate and stepwise drying (70 °C for 3 h, 170 °C for 3 h, and 220 °C for 1 h, respectively) to allow for the slow evaporation of DMAc. The films were stripped off the substrates and further dried in a convection oven at 70 °C for 6 h.

### 2.5. Characterization

The molecular weights of SHPIs were determined by gel permeation chromatography (GPC) on a 1260 Infinity II instrument (Agilent, Santa Clara, CA, USA) using DMF as an eluent at a concentration of 1 mg/mL. FTIR spectra of SHPIs were obtained on a NICOLET iS50 spectrometer (Thermo Fisher, Waltham, MA, USA) with an ATR accessory. ^1^H NMR spectra of SHPIs dissolved in DMSO-d_6_ were recorded on a Bruker DRX 400 (400 MHz) spectrometer (Bruker, Karlsruhe, Germany). X-ray diffraction (XRD) was carried out on a Smartlab (9 kW) (Rigaku, Tokyo, Japan) with Cu Kα radiation (λ = 0.15405 nm), and the accelerating voltage and emission current were 40 kV and 200 mA, respectively. UV–Vis spectra were recorded on a U-3900 spectrometer (Hitachi, Tokyo, Japan) in transmittance mode at 1 nm intervals from 200 to 800 nm. Thermal properties were measured using thermogravimetric analysis (TGA) and derivative thermogravimetry (DTG) on a TG209 F3 thermal analyzer (Netzsch, Selb, Germany). Approximately 5–10 mg of each sample was heated from 30 °C to 800 °C at 10 °C min^−1^ under an argon atmosphere flowing at 50 mL min^−1^. Differential scanning calorimetry (DSC) was carried out on a Q20 instrument (TA, Salt Lake City, UT, USA) from 0 to 300 °C with heating and cooling at 10 °C min^−1^ under a nitrogen atmosphere. Dynamic mechanical analysis (DMA) was carried out on a Q800 instrument (TA, USA) from 35 °C to 300 °C at a heating rate of 3 °C min^−1^ and a frequency of 1 Hz. X-ray photoelectron spectroscopy (XPS) was carried out on a Thermo Scientific K-Alpha instrument (Thermo Fisher, USA). Surface morphology was observed using a JSM-7200F scanning electron microscope (SEM, JEOL, Akishima, Japan). Elemental compositions were determined using energy dispersive spectroscopy (EDS, NORAN System 7, Thermo Scientific, USA). The samples were coated with a thin gold layer.

To test the self-healing performance, SHPI films were scratched using a DN-52 cutter blade (0.2 mm in thickness, DORCO, Seoul, Republic of Korea) under a constant load (100 g) on a DHZ scratch tester (Jingkelian Material Testing Machine, Tianjin, China), followed by healing in an oven under different conditions. The self-healing behaviors of scratched surfaces of SHPI films were monitored using a BX41 polarizing microscope (OLYMPUS, Tokyo, Japan). An AI-7000M tensile testing machine (Gotech, Dongguan, China) was used to evaluate the healing efficiencies of scratched SHPI films. The specimens were cut into small rectangular strips, with a dimension of 50 × 10 × 0.08 mm^3^, and each sample was subjected to 3 individual tensile tests. The self-healing efficiency (η) was calculated using Equation (1) [28,29], as follows:

(1) $$\eta (\%)=\frac{\sigma_{h}}{\sigma_{0}}\times 100\%$$

where σ_0_ and σ_h_ are the tensile strengths of the pristine and healed films, respectively.

The stress relaxation behaviors of the SHPI films were measured using a TA Q800 Instrument. The samples were initially applied at a preload force of 0.001 N to maintain straightness. After thermal retardation at different testing temperatures, a strain of 2% was exerted within the linear region of the material and the relaxation modulus was collected as a function of time.

## 3. Results and Discussion

### 3.1. Structure and Properties of SHPI Films

Although the molecular weights of SHPIs showed a decreasing trend with increasing contents of cystamine (Table 1), all samples had relatively high molecular weights (M_n_ > 9 × 10^4^ g/mol) and low polydispersity indices (PDI < 1.71). In fact, due to strong basicity, aliphatic diamines are easily ionized during dissolution. The ionized aliphatic diamines tend to form ‘salt bridges’ with polyamic acid (PAA) at the beginning of polymerization, thus interfering with the increase in molecular weights. However, the main chain structure of SHPIs contained various flexible linkages, such as -O-, -S- and -CH_2_- groups, as well as bulky -CF_3_ groups, which increased the intermolecular free volume and inhibited the formation of heterogeneous salts, resulting in a relatively high molecular weight [30,31]. The molecular weights, structures, and properties of the other formulated samples (SHPI-0.2, SHPI-0.4 and SHPI-0.6) are detailed in the Supplementary Materials (Part B and C in the Supplementary Materials).

**Figure 1:**
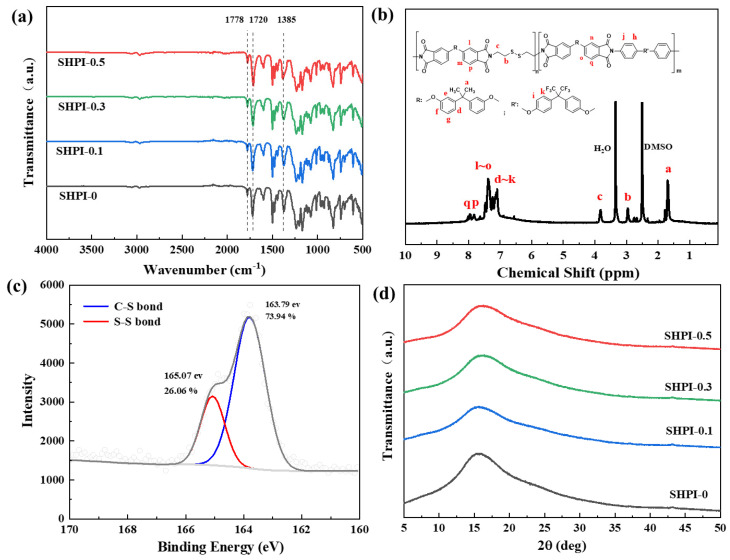
Structural properties of SHPI films: (**a**) FT-IR spectra; (**b**) H^1^ NMR spectrum of SHPI-0.5; (**c**) high-resolution S 2p XPS spectra of SHPI-0.5; and (**d**) XRD spectra.

In the FT-IR spectra of the SHPIs (Figure 1a), three characteristic absorption peaks at 1778 cm^−1^, 1720 cm^−1^ and 1385 cm^−1^ originated from the asymmetric stretching of C=O, the symmetric stretching of C=O, and the stretching vibration of C-N-C, respectively, confirming the formation of imides. No peaks of isoimides were attributed to the carbonyl stretching observed at 1795–1820 cm^−1^ or 921–934 cm^−1^, indicating the absence of isoimides. In addition, no peaks near 1670 cm^−1^ corresponding to the carbonyl stretching of an intermolecular imide linkage were observed [32]. These results indicated that the SHPIs were fully imidized.

The chemical structure of the SHPIs was confirmed using ^1^H NMR. Figure 1b shows the ^1^H NMR spectrum of SHPI-0.5 as an example in which each hydrogen proton was correspondingly assigned. The absorptions were divided into two regions: the downfield one caused by aromatic rings and the upfield one caused by the alkyl chain. The peaks at 2.95 ppm and 3.82 ppm are attributed to hydrogen protons in the two methylene groups in the cystamine structural fragments, respectively, indicating the successful preparation of the SHPIs. The reversible covalent bonding of the disulfide functional group in SHPI-0.5 was investigated using XPS. The characteristic peaks at 688, 535, 400, 285, and 164 eV were attributed to fluorine (1s), oxygen (1s), nitrogen (1s), carbon (1s), and sulfur (2p) [33], respectively (Figure S2). In addition, in the high-resolution S 2p XPS spectra (Figure 1c), there were two characteristic peaks at 163.79 and 165.07 eV, which corresponded to two functional groups of the polyimide chain, i.e., the S-C and S-S bonds, respectively [34].

In the wide-angle X-ray diffraction pattern of the SHPIs (Figure 1d), there were broad peaks centered at 2θ = 16° due to the diffraction of intermolecular packing, which is a characteristic of amorphous materials [32]. This result indicated that the SHPIs possessed an amorphous structure owing to the loose chain packaging and aggregation caused by the ether and isopropylidene linkages in dianhydride, as well as to the trifluoromethyl groups (-CF_3_) in diamine.

**Figure 2:**
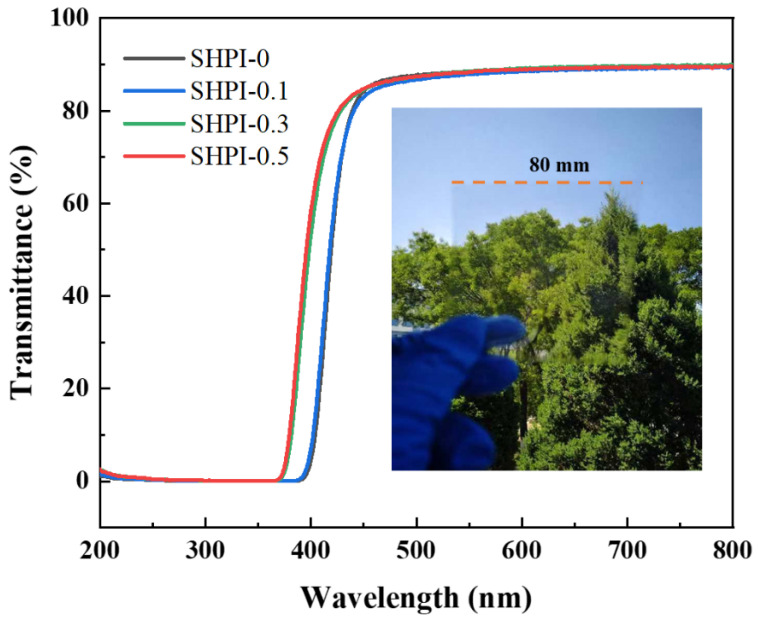
UV–Vis spectra of SHPI films. The inserted image shows the excellent optical transparency of the SHPI films.

The optical transparency of SHPI films was evaluated by UV–Vis spectroscopy. As shown in Figure 2, the 80 µm-thick SHPI films showed very high optical transparency, with cut-off wavelengths (λ_cut_) below 400 nm, a transmittance, at 500 nm (T_500nm_), above 87%, and a yellowness index (YI) below 10, possibly due to decreased charge transfer (CT) and electronic conjugation in the PI chain structures with the increasing contents of aliphatic diamines [30]. Meanwhile, the highly electronegative -CF_3_ groups could efficiently reduce the CT from electron-donating diamines to electron-withdrawing dianhydrides, thereby prohibiting visible light absorption by the polymers [35].

**Figure 3:**
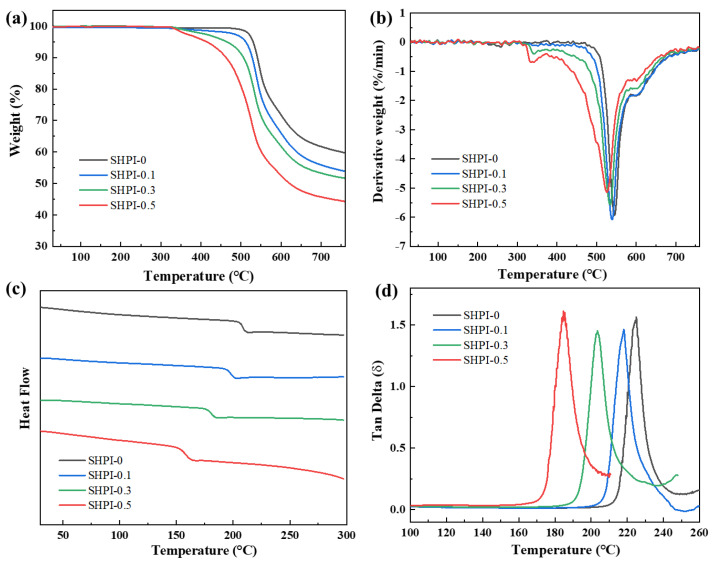
Thermal properties of SHPI films: (**a**) TGA curves; (**b**) DTG curves; (**c**) DSC curves; and (**d**) DMA curves.

The thermal decomposition behaviors of SHPI films were investigated using TGA and DTG; the results are presented in Figure 3a,b and Table 2, respectively. In contrast to SHPI-0, the weight loss of SHPIs containing both cystamine and HFBAPP (SHPI-0.1, SHPI-0.3, and SHPI-0.5) occurred in two stages. The first stage occurred at around 300–400 °C and was involved with the S-S and S-C bonds in the repetitive unit consisting of cystamine and BPADA, which are less stable than the C-C or C-O bonds at high temperatures [36,37]. The second stage of decomposition is related to the repetitive unit formed by the condensation of HFBAPP and BPADA, similar to that of the ordinary film (SHPI-0) [24]. Although the onset decomposition temperature, at a mass loss of 5% (T_5%_) for the SHPI films, decreased with the introduction of cystamine, the SHPI films with lower contents (≤50%) of cystamine had higher decomposition temperatures (T_5%_ ≥ 415 °C), with other reported results shown in Table S3 (Supplementary Materials).

The glass transition behaviors of the SHPI films were observed using DSC and DMA. The DSC curves clearly showed that the SHPI films exhibited typical glass transition behaviors (Figure 3c). It should be noted that the T_g, DSC_ values of the SHPI films with the addition of cystamine (SHPI-0.1, SHPI-0.3, and SHPI-0.5) were lower than that of the ordinary SHPI film (SHPI-0) without cystamine and that they tended to decrease with increasing contents of cystamine. Similar results were also observed in T_g,DMA_ values identified as the peak temperatures in the DMA curves (Figure 3d). The T_g,DMA_ values of the copolymer SHPI films (SHPI-0.1, SHPI-0.3, and SHPI-0.5) ranged between 184 and 218 °C, which were also relatively lower than those of the SHPI-0 (Table 2). The changes in T_g,DSC_ and T_g,DMA_ indicate that the introduction of cystamine with an aliphatic chain structure remarkably improved the flexibility of the polymer backbone. In general, self-healing materials tend to have a low T_g_ to ensure the sufficient motility of molecular chains for self-healing at low temperatures [23,24]. Although the incorporation of cystamine reduced the T_g_ of SHPI films, it enhanced their self-healing ability by introducing dynamic covalent disulfide bonds and flexible methylene groups. Therefore, it is crucial to balance the thermal and self-healing properties by controlling the monomer structure and the ratios of HFBAPP and cystamine.

### 3.2. Self-Healing Behaviors of SHPI Films

**Figure 4:**
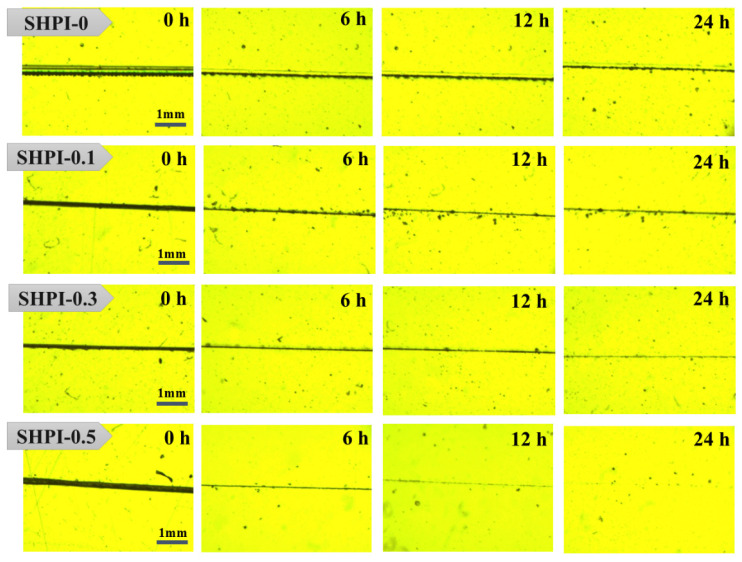
Self-healing processes of scratched SHPI films observed using an BX41 polarizing microscope.

**Figure 5:**
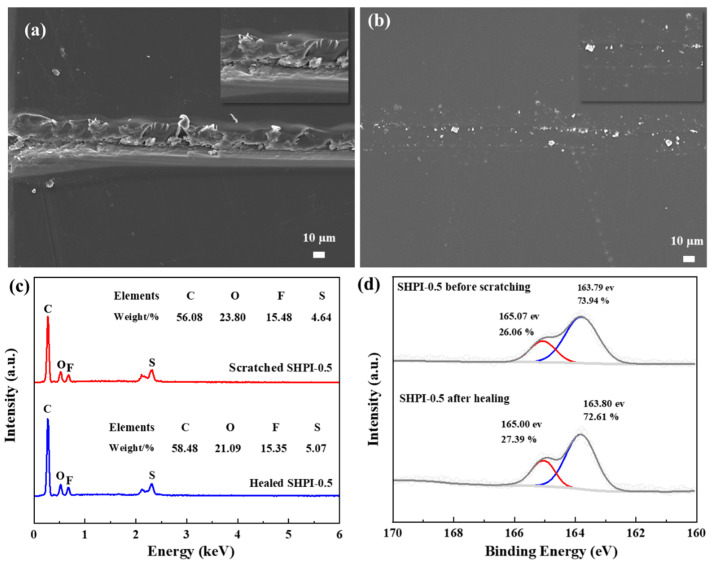
SEM images of SHPI-0.5 films: (**a**) scratched film; (**b**) healed film; (**c**) EDS spectra of scratched and healed SHPI-0.5 films; and (**d**) high-resolution S 2p XPS spectra of SHPI-0.5 films before scratching and after healing.

The self-healing behavior of SHPI films was investigated using polarized light microscopy and tensile tests. The initial scratch depth was set to half the film thickness, and a constant load was applied to the surface of SHPI films to produce a zigzag scratch. After heat treatment at the healing temperature (T_g,DSC_ + 30 °C) for 6 h, 12 h, and 24 h, the self-healing behaviors of scratched films were observed using an optical microscope with a hot stage. As shown in Figure 4, after heat treatment at T_g,DSC_ + 30 °C for 6 h, the scratches on all SHPI films diminished, with SHPI-0.5 showing the fastest healing rate. After 24 h of heat treatment, the scratch on SHPI-0.5 films had been completely healed, while the scratches on other films (SHPI-0.3, SHPI-0.1 and SHPI-0) were still visible, with SHPI-0.3 < SHPI-0.1 < SHPI-0 in terms of the visibility of scratches. These results suggest that the self-healing ability of SHPI films could be substantially improved by increasing the contents of the disulfide bonds. The SEM images of SHPI-0.5 films showed that the pristine film was 80.3 μm in thickness, and the depth of the scratch was 40.7 µm (Figure 5a and Figure S4a); after heat treatment at 190 °C for 24 h, the scratches had been almost completely healed (Figure 5b and Figure S4b). The EDS spectra showed that the sulfur contents on the surfaces of the scratched and healed films were almost the same (Figure 5c), indicating no significant weight loss during the self-healing process. Moreover, there were no significant changes in the high-resolution S 2p XPS spectra between the scratched and healed SHPI-0.5 films (Figure 5d), indicating no chemical changes in the film during the self-healing process through disulfide exchange reactions. In other words, the self-healing process did not affect the chemical bonds and structure of the SHPI films, which enabled the film to repeatedly heal the scratches in the same position [38].

**Figure 6:**
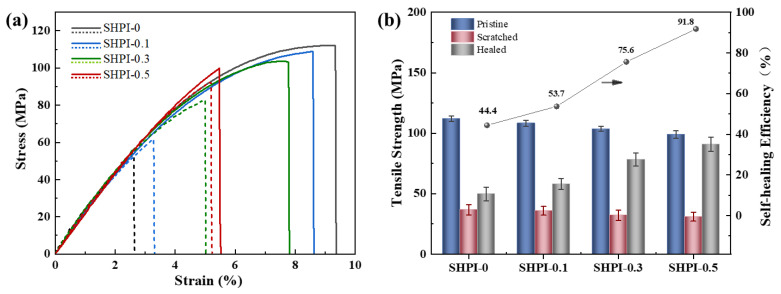
Mechanical properties and healing capacities of SHPI films: (**a**) stress–strain curves of SHPI films (bold line: pristine films; dashed line: healed films); and (**b**) tensile strength and self-healing efficiencies of SHPI films.

Tensile tests were carried out to quantitatively evaluate the mechanical properties and healing capacities of the materials. As shown in Figure 6 and Table 3, the addition of cystamine decreased the tensile strengths of the SHPI films, which was consistent with the changes in the molecular weight of the SHPIs (Table 1). On the one hand, when the content of cystamine was increased, the content of HFBAPP was also decreased, further leading to the reduced amount of rigid benzene rings and -CF_3_ in the structure of the SHPIs. In this way, the tensile strength of the final material deteriorated to some extent [23]. In addition, the decrease in molecular weight caused by the addition of cystamine is one of the important reasons contributing to the weakening of the mechanical properties of the films. When the content of cystamine reached 50%, the tensile strength of SHPI-0.5 was about 99 MPa, which was higher than those of most reported self-healing PI materials (Table S3 in Supplementary Materials). After heat treatment at T_g,DSC_ + 30 °C for 24 h, the self-healing efficiency of the SHPI films significantly increased with increasing contents of cystamine (Table 3) and the self-healing efficiency of the SHPI-0.5 film reached 91.8%, indicating that it had the highest self-healing ability. In general, self-healing polymers are able to repair the damage through chain reorganization under external physical stimuli (such as heat, electricity, and UV). However, two conditions must be fulfilled: the presence of elements capable of restoring intra-chain bonds or attraction; and the sufficient mobility of polymer chains [24]. In the present study, as the content of cystamine increased, there were sufficient dynamic S-S bonds in the polymer chains to undergo exchange reactions in the scratched region under heat treatment, leading to self-healing through molecular chain reorganization and network cross-linking [35,39]. In addition, with the decreased content of HFBAPP, the amounts of -CF_3_ and benzene rings in the structure decreased; however, the amount of -CH_2_ increased, resulting in the increased flexibility and motility of the molecular chain. Therefore, the fulfillment of these two conditions remarkably improved the self-healing performance of the SHPI films.

### 3.3. Stress Relaxation Behaviors of SHPI Films

**Figure 7:**
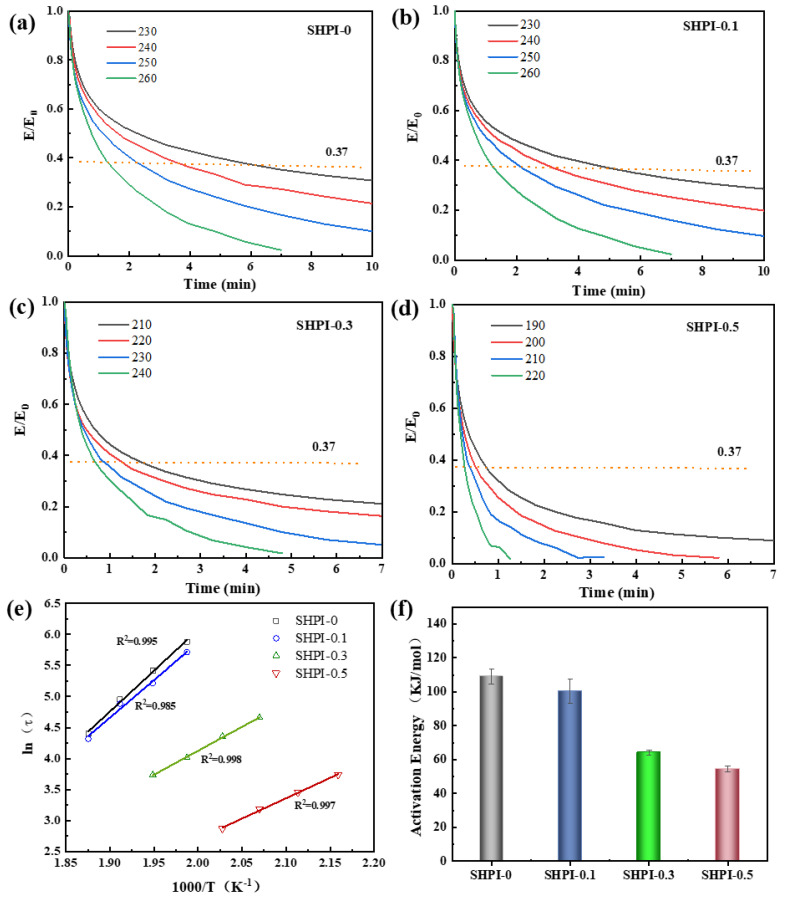
Stress relaxation behaviors of SHPI films. Normalized stress-relaxation curves of: (**a**) SHPI-0; (**b**) SHPI-0.1; (**c**) SHPI-0.3; and (**d**) SHPI-0.5 films at different temperatures. (**e**) The Arrhenius analysis of SHPI films; and (**f**) activation energy of SHPI films.

We further observed the effects of the introduction of dynamic disulfide bonds on the stress relaxation behaviors of the SHPI films at different temperatures. The relaxed tensile modulus was recorded over time at different temperatures under a constant strain of 2%. The relaxation time (τ) is defined as the time spent for SHPI films to relax to 1/e (i.e., 37%) of the initial modulus [40]. As shown in Figure 7a–d, compared to other SHPI films, the chain backbone of SHPI-0.5 relaxed more rapidly at temperatures slightly above T_g, DMA_ (184 °C). Its relaxation time at 190 °C was about 45 s and decreased with increasing temperatures, indicating that disulfide bonds were cleaved more rapidly and that the network topology was transformed at higher temperatures [41,42,43].

Figure 7e shows that the relaxation time as a function of temperature could be described by the Arrhenius equation (Equation (2)) [43], as follows:

(2) $$\tau \left(T\right)=\tau_{0}\exp\left(\frac{E_{a}}{RT}\right)$$

where *τ*_0_ is the relaxation time at the infinite temperature, *R* is the universal gas constant, *T* is the temperature, and *E_a_* is the activation energy. Based on this equation, the activation energies (*E_a_*) were calculated to be 109.16, 100.61, 64.27 and 54.59 kJ/mol for SHPI-0, SHPI-0.1, SHPI-0.3, and SHPI-0.5, respectively. In fact, a significant number of molecular chain migration, diffusion, and reversible bond exchange reactions occur during the relaxation process of self-healing polymers, which indirectly reflects their healing ability [42]. The activation energy is calculated by the Arrhenius equation to display the energy needed for the fracture and generation of dynamic bonds in the self-healing polymers [44]. The lower activation energies could facilitate the topological rearrangements due to the rapid exchange reactions, leading to a superior self-healing ability [45,46].

## 4. Conclusions

In summary, this study reported a technological approach to preparing a self-healing transparent polyimide (SHPI) film using cystamine as a functional monomer through copolymerization by adjusting the ratios of HFBAPP and cystamine. The results showed that the self-healing ability of the SHPI films increased significantly with the increasing contents of cystamine, and that SHPI-0.5 exhibited the best self-healing performance, with a repair efficiency as high as 91.8%. The stress relaxation results showed that SHPI-0.5 had a short relaxation time (45 s) at 190 °C, and that the low relaxation activation energy (54.59 kJ/mol) facilitated the topological rearrangements due to rapid exchange reactions. In addition, the incorporation of cystamine could maintain the good optical properties of SHPIs, but it also decreased the mechanical properties and thermal stability to some extent. SHPI-0.5 exhibited a high transmittance of 87.46% at 500 nm, a tensile strength of 99 MPa, and an onset of thermal decomposition temperature of 415 °C, which is still advantageous in terms of its comprehensive properties compared with reported self-healing PI materials. The integration of unusual optical properties with self-healing abilities guarantees the potential applications of SHPIs in advanced optoelectronic devices.

## Figures and Tables

**Table 1:** Composition and molecular weights of SHPIs.

Samples	BPADA (mmol)	HFBAPP (mmol)	Cystamine (mmol)	M_n_ (10^4^ g/mol)	M_w_ (10^4^ g/mol)	PDI
SHPI-0	5	5	0	16.71	25.58	1.53
SHPI-0.1	5	4.5	0.5	16.14	26.02	1.61
SHPI-0.3	5	3.5	1.5	13.78	23.02	1.67
SHPI-0.5	5	2.5	2.5	9.03	15.50	1.71

M_n_: number average molecular weights, M_w_: weight average molecular weights, PDI: polydispersity index, and PDI = M_w_/M_n_.

**Table 2:** Optical and thermal properties of SHPI films.

Samples	λ_cut_ (nm)	T_500nm_ (%)	YI	T_5%_ (°C)	T_g,DSC_ (°C)	T_g,DMA_ (°C)
SHPI-0	389	87.75	11	530	204	225
SHPI-0.1	386	87.02	8.5	511	195	218
SHPI-0.3	368	87.42	7.7	464	181	203
SHPI-0.5	367	87.46	6.1	415	160	184

λ_cut_: cut-off wavelengths; T_500nm_: the transmittance at 500 nm; YI: yellowness index; T_5%_: onset decomposition temperature at a mass loss of 5%; T_g,DSC_ and T_g,DMA_: glass transition temperatures determined using DSC and DMA, respectively.

**Table 3:** Mechanical properties and self-healing efficiency of SHPI films.

Samples		Tensile Strength (MPa)	Young’s Modulus (GPa)	Elongation at Break (%)	Self-Healing Efficiency (%)
SHPI-0	Pristine	112.01 ± 2.29	2.28 ± 0.14	9.43 ± 0.17	44.4
	Scratched	36.67 ± 4.17	2.21 ± 0.19	1.71 ± 0.19	
	Healed	49.70 ± 5.59	2.29 ± 0.14	2.41 ± 0.23	
SHPI-0.1	Pristine	108.18 ± 2.57	2.19 ± 0.12	8.57 ± 0.29	53.7
	Scratched	35.81 ± 3.54	2.16 ± 0.13	1.65 ± 0.21	
	Healed	58.11 ± 4.64	2.27 ± 0.12	3.02 ± 0.31	
SHPI-0.3	Pristine	103.54 ± 2.03	2.25 ± 0.14	7.79 ± 0.30	75.6
	Scratched	32.25 ± 4.30	2.19 ± 0.15	1.47 ± 0.22	
	Healed	78.33 ± 5.47	2.47 ± 0.15	4.99 ± 0.15	
SHPI-0.5	Pristine	98.96 ± 2.96	2.11 ± 0.09	5.75 ± 0.19	91.8
	Scratched	31.01 ± 3.76	2.10 ± 0.11	1.45 ± 0.18	
	Healed	90.83 ± 5.85	2.24 ± 0.12	5.19 ± 0.30	

## Data Availability

The original contributions presented in this study are included in the article/Supplementary Material. Further inquiries can be directed to the corresponding author.

## Supplementary Materials

The following supporting information can be downloaded at: https://www.mdpi.com/article/10.3390/polym16243461/s1. Figure S1. Characterization of cystamine: (a) FTIR spectra; (b) H^1^ NMR spectra; and (c) C^13^ NMR spectra. Cystamine was dissolved in DMSO-d_6_, and the measurements were performed at room temperature. Figure S2. Characterization of SHPI films: (a) FTIR spectra; (b) XRD spectra; and (c) full XPS spectra of SHPI-0.5 film. Figure S3. Properties of SHPI films: (a) UV–Vis spectra; (b) TGA curves; (c) DTG curves; (d) DSC curves; (e) DMA curves; and (f) stress–strain curves. Figure S4. SEM images of the cross-sections of SHPI-0.5 films: (a) scratched films; and (b) self-healed films. Table S1: composition and molecular weights of SHPIs. Table S2: properties of SHPI films. Table S3: Performance parameters of self-healing polyimides.
