# Supplementary material for: Preparation and Characterization of a Novel Self-Healing Transparent Polyimide Film Based on Dynamic Disulfide Bonds
Source: Polymers (Basel). 2024 Dec 11;16(24):3461. doi: 10.3390/polym16243461 (PMC11728536; doi:10.3390/polym16243461)
Supplement: Supplementary file 1 [file polymers-16-03461-s001.zip › polymers-3338334-supplementary.pdf]

Supplementary material

Preparation and characterization of a novel self-healing transparent polyimide film based on dynamic disulfide bonds

Xin Li, Yan Zhai*, Kai Yang, Jingjing Bai, Yu Qiu, Yulong Wang

Department of Materials Engineering, Taiyuan Institute of Technology,

Taiyuan, Shanxi 030008, China

*Corresponding authors. E-mail: zhaiy@tit.edu.cn

Part A: Characterization of cystamine

As shown in Figure S1, the structure of cystamine was characterized using FTIR, ^{1}H NMR and ^{13}C NMR.

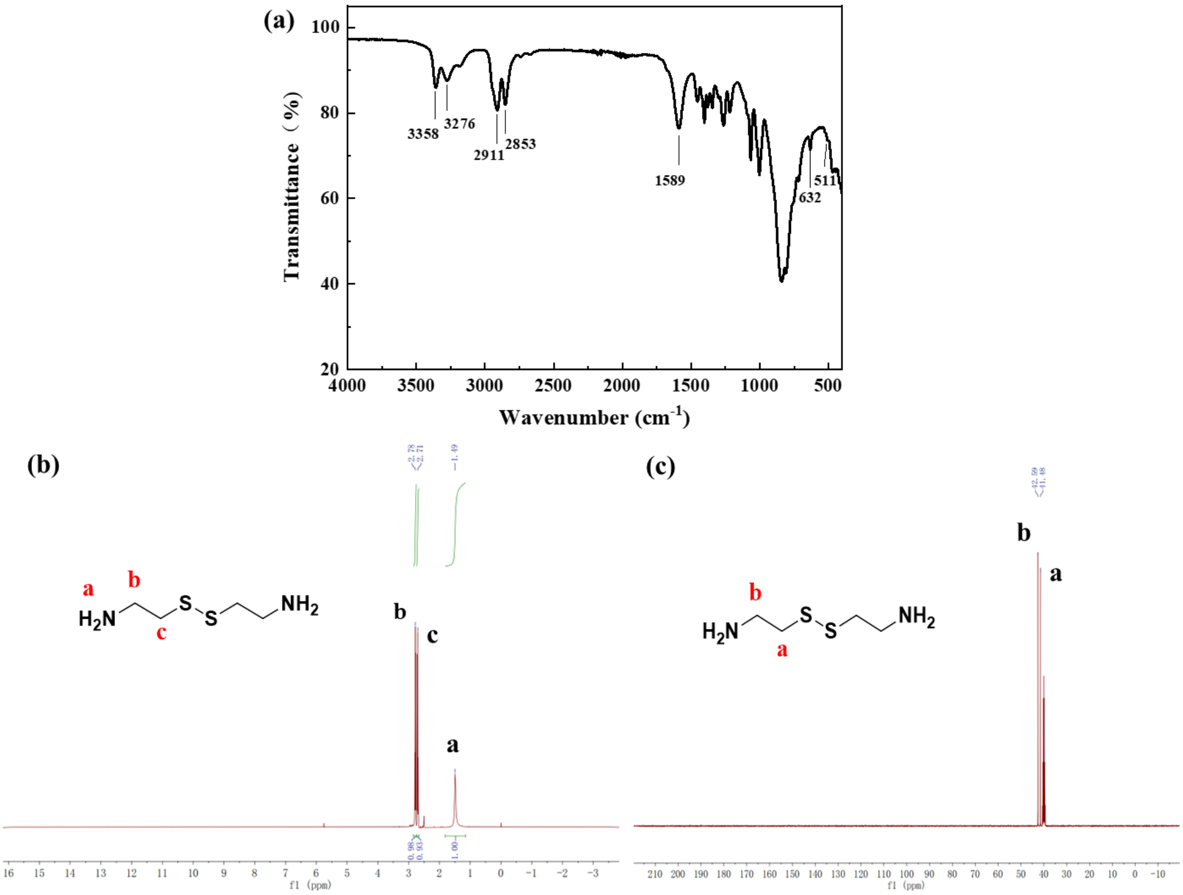

Figure S1 Characterization of cystamine. (a) FTIR spectra, (b) H^1 NMR spectra, and (c) C^{13} NMR spectra. Cystamine was dissolved in DMSO-d_6, and the measurements were performed at room temperature.

Part B: Characterization of SHPI films

As shown in Figure S2, the molecular weights and structures of SHPI films prepared at monomer ratios of 0.2, 0.4 and 0.6 were characterized using GPC, FTIR, XRD and XPS, and the results are shown in Table S1.

Table S1. Composition and molecular weights of SHPIs

Samples	BPADA (mmol)	HFBAPP (mmol)	Cystamine (mmol)	M_n (10^4 g/mol)	M_w (10^4 g/mol)	PDI
SHPI-0.2	5	4	1	15.17	23.92	1.57
SHPI-0.4	5	3	2	10.51	16.91	1.61
SHPI-0.6	5	2	3	7.86	12.64	1.60

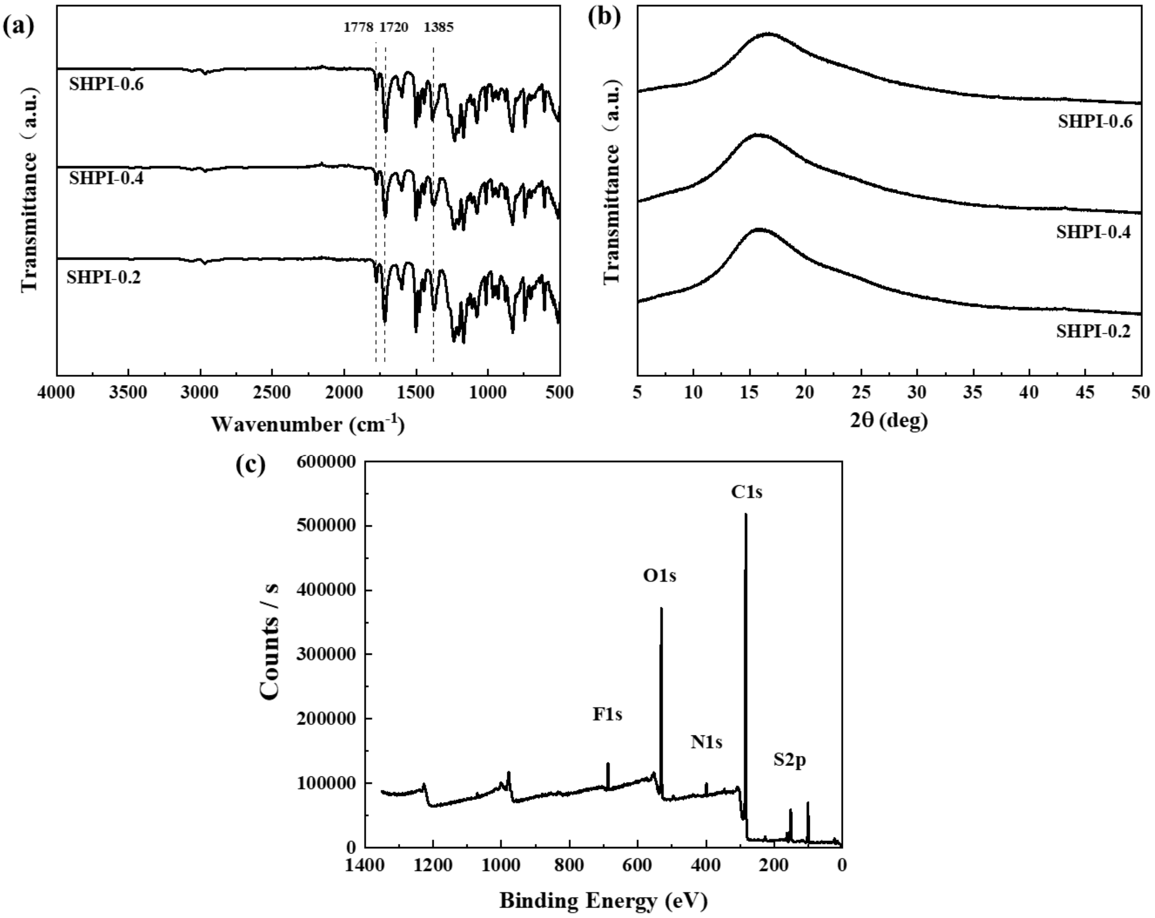

Figure S2 Characterization of SHPI films. (a) FTIR spectra, (b) XRD spectra, and (c) Full XPS spectra of SHPI-0.5 film

Part C: Properties of SHPI films

Optical, thermal and mechanical properties of SHPI films were systematically investigated and the obtained original spectra are shown in Figure S3(a-f). The results are shown in Table S2.

Table S2. Properties of SHPI films

Sample	λ_{cut} （nm）	T_{500nm} (%)	YI	$T_{5\%}$ (°C)	$T_{g, DSC}$ (°C)	$T_{g, DMA}$ (°C)	σ (MPa)
SHPI-0.2	368	87.51	7.8	495	184	208	106.15±3.10
SHPI-0.4	363	86.79	6.9	427	165	194	99.29±2.90
SHPI-0.6	370	84.40	8.1	394	151	174	50.08±3.38

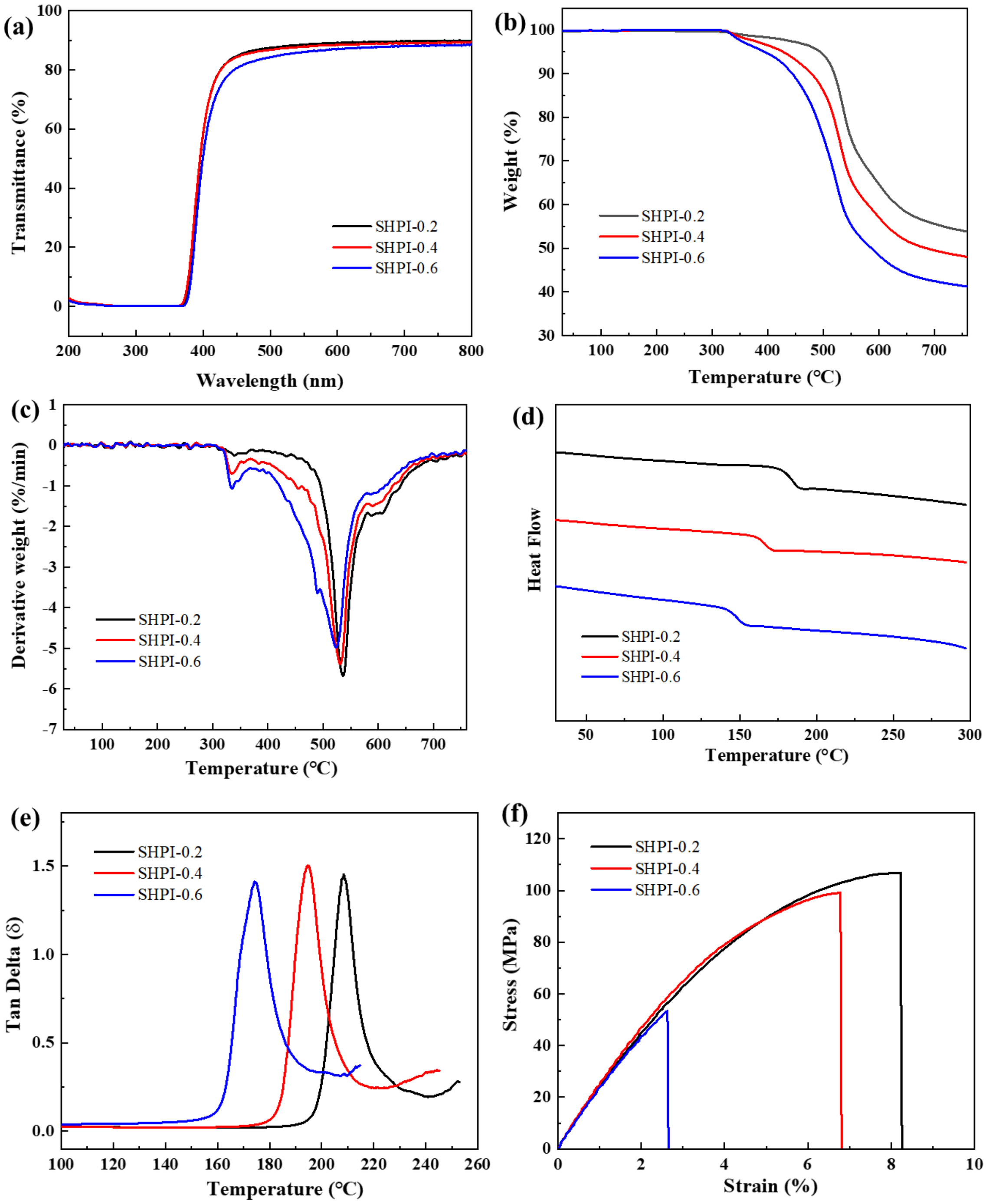

Figure S3 Properties of SHPI films. (a) UV-Vis spectra, (b) TGA curves, (c) DTG curves, (d) DSC curves, (e) DMA curves, and (f) Stress-strain curves

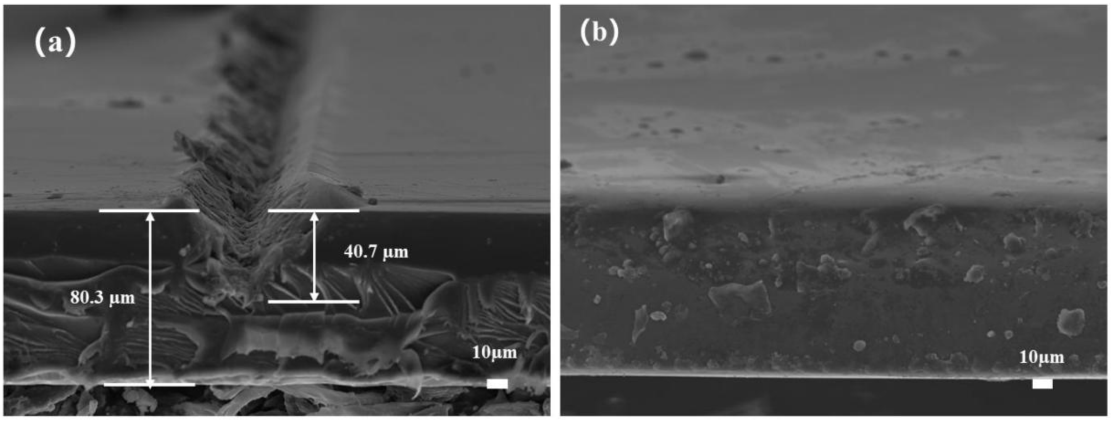

Figure S4. SEM images of the cross-sections of SHPI-0.5 films: (a) Scratched films, and (b) Self-healed films.

Table S3. Performance parameters of self-healing polyimides

Samples	T_g (℃)	$T_{5\%}$ (℃)	Tensile strength (MPa)	Self-healing efficiency (%)	$T_{500\ nm}$ (%)	Ref.
PBF 5 wt. %	—	350	6.8	91	72	[1]
BPADA-D	13	380	8	99	—	[2]
APD:BAPB=10:0	190	490	38	92	—	[3]
6FDA14	95	330	57.58	98	95	[4]
PUPI	71.3	277.8	70	99	—	[5]
6B19	108	450	77	96.7	90	[6]
SHSMPI	218	450	117	86	—	[7]
SHPI	160	415	99	91.8	87.46	Our work

References

1. Kim Y. N., Nam K. H., Yong. C J., Han H. Interfacial adhesion and self-healing kinetics of multi-stimuli responsive colorless polymer bilayers. Composites Part B. 2020,203, 108451. https:// doi.org/10.1016/j.compositesb.2020.108451.
2. Susa A, Mordvinkin A, Saalw achter K, Van Der Zwaag S, Garcia SJ. Identifying the role of primary and secondary interactions on the mechanical properties and healing of densely branched polyimides. Macromolecules. 2018, 51, 8333. https:// doi.org/ 10.1021/acs.macromol.8b01396.
3. Wan B. Q., Dong X. D., Yang X., Zheng M. S., Chen G., Zha J. W. High strength, stable and self-healing copolyimide for defects induced by mechanical and electrical damages. Journal Materials Chemistry C. 2022, 10, 11307-11315. https:// doi.org/10.1039/ D2TC01605B.
4. Kim Y. N., Lee J., Kim Y., Kim J., Han H., Jung Y. C. Colorless polyimides with excellent optical transparency and self-healing properties based on multi-exchange dynamic network. Applied Materials Today. 2021,25, 101226. https:// doi.org/ 10.1016/j. apmt.2021.101226
5. Lee S., Hong P. H., Kim J., Choi K., Moon G., Kang J., Lee S., Ahn J. B., Eom W., Ko M. J., Hong S. W. Highly Self-Healable Polymeric Blend Synthesized Using Polymeric Glue with Outstanding Mechanical Properties. Macromolecules. 2020, 53, 6, 2279-2286. https:// doi.org/10.1021/acs.macromol.9b02359.
6. Kim Y. N., Jo J. Y., Park J., Lee J., Kim J., Jeon D., Han H., Jung Y. C. Challenge for Trade-Off Relationship between the Mechanical Property and Healing Efficiency of Self-Healable Polyimide. ACS Appl. Mater. Interfaces 2023, 15, 54923-54932. https:// doi.org/10.1021/acsami.3c12594.
7. Kong D.Y., Li J., Guo A. R., Zhang X. T., Xiao X. L. Self-healing high temperature shape memory polymer. European Polymer Journal. 2019, 120, 109279. https:// doi.org/10.1016/j.eurpolymj.2019.109279.
